# Brachyury: A Diagnostic Marker for the Differential Diagnosis of Chordoma and Hemangioblastoma versus Neoplastic Histological Mimickers

**DOI:** 10.1155/2014/514753

**Published:** 2014-01-21

**Authors:** Valeria Barresi, Antonio Ieni, Giovanni Branca, Giovanni Tuccari

**Affiliations:** ^1^Department of Human Pathology “G. Barresi”, University of Messina, Italy; ^2^Dipartimento di Patologia Umana, Azienda Ospedaliera Universitaria “Policlinico G. Martino”, Pad. D, Via Consolare Valeria 1, 98125 Messina, Italy

## Abstract

Brachyury is a transcription factor which is required for posterior mesoderm formation and differentiation as well as for notochord development during embryogenesis. Due to its expression in the neoplastic cells of chordoma, a malignant tumour deriving from notochordal remnants, but not in tumors showing a similar histology, brachyury has been proposed as a diagnostic marker of this neoplasia. Though commonly considered a hallmark of chordoma, the expression of brachyury has been also documented in the stromal cells of hemangioblastoma (HBL), a slow growing tumor which may involve the central nervous system (CNS) and, rarely, the kidney. Herein we review the role of brachyury immunohistochemical detection in the identification and differential diagnosis of chordoma and HBL towards histological mimickers and suggest that brachyury is added to the panel of immunohistochemical markers for the recognition of HBL in routinary practice, principally in unusual sites.

## 1. Introduction

Brachyury is a transcription factor encoded by T, a member of the T-box gene family, and required for posterior mesoderm formation and differentiation [1] as well as for notochord development [2]. In accordance with its function, brachyury is expressed in all nascent mesoderm [2, 3], in the embryo. Along with embryonic differentiation, it is downregulated [2, 3], restricted into the notochord and the tail bud [1], and then lost, when notochordal cells are replaced by bone in the vertebral bodies and by the nucleus pulposus in the intervertebral discs [4]. In the adult, brachyury expression has been found in chordoma [4], a malignant tumor which recapitulates notochord and derives from small collections of notochordal cells which may persist into the adult life [5]. Several studies have shown that brachyury represents a specific marker for chordoma, useful to discriminate this neoplasia from others with a similar histology [4–12]. Nonetheless, there is evidence that chordoma is not the only tumour expressing brachyury. Indeed, the expression of this protein has been also documented in hemangioblastoma (HBL) [6, 7, 13, 14], a slow growing vascular tumour, which origins from mesoderm derived, embryologically arrested hemangioblasts [4, 13], which also express this protein [15].

Herein, the use of brachyury immunohistochemical staining for the differential diagnosis of chordoma and HBL towards neoplastic histological mimickers occurring in the same sites is discussed.

## 2. Brachyury for the Differential Diagnosis of Chordoma

Chordoma is an intraosseous, low to intermediate grade, malignant tumor with a tendency to recur or metastasize [16]. Being derived from notochordal remnants [5], the histologic aspect of chordoma reminds notochordal structures and this tumor mainly localizes at the sacrococcygeal, spheno-occipital and vertebral regions, where embryological residues of the notochord are more commonly found [17]. Due to their proximity to the meninges, chordomas located in the spheno-occipital and vertebral regions may secondarily invade the dura. However, primary intradural chordomas without bone infiltration [6, 18, 19], displaying a more favourable course compared to intraosseous chordomas [20], have been rarely reported. Finally, a small number of tumors, showing an identical morphology to axial chordomas, have been also described as primary extra-axial or soft tissues chordomas [21].

Morphologically, chordoma is composed of epithelioid cells showing prominent vacuoles—giving it the characteristic physaliphorous appearance—and arranged in cords within a myxoid to chondromyxoid matrix. Several histotypes of this tumour may be recognized based on the aspect of the cells and intervening matrix, including classical chordoma, chordoma with a dominant chondroid component, chondroid chordoma, and dedifferentiated chordoma [22]. Owing to its histological aspect, chordoma needs to be differentiated from many other tumors, including chondrosarcoma, metastatic adenocarcinoma, clear cell renal cell carcinoma (CCRCC), or central nervous system (CNS) neoplasias with a chordoid appearance.

The principal differential diagnosis of chordoma is versus chondrosarcoma. Differentiating the two lesions is clinically relevant, as low grade chondrosarcoma is treated with conservative surgery, whereas chordoma often requires adjuvant radiotherapy in view of its tendency for recurrence and metastasis. A significant help to solve the diagnostic dilemma comes from immunohistochemistry. Indeed, chordoma was originally described as one of the unique “triple positive” EMA/S100 protein/keratins neoplasia in bone and soft tissue pathology [23] and diffuse immunostaining for wide spectrum cytokeratins, cytokeratin-8, cytokeratin-19, and cytokeratin-18 was demonstrated in this tumour [24]. Though in most of the cases a definitive diagnosis may be established by using a panel including keratins and EMA—positive in chordoma and negative in chondrosarcoma—it may still be hard to differentiate between chordoma and chondrosarcoma in needle core biopsies based on keratins stain only, since cytokeratin expression may not be present throughout the chondroid component of chondroid chordoma [25]. In addition, immunohistochemistry against cytokeratins leaves unsolved the differential diagnosis of chordoma towards other mimickers, such as metastatic mucinous adenocarcinoma, salivary gland carcinoma (head and neck region), myoepithelial tumors, metastatic renal cell carcinoma, or seminoma.

**Figure 1:**
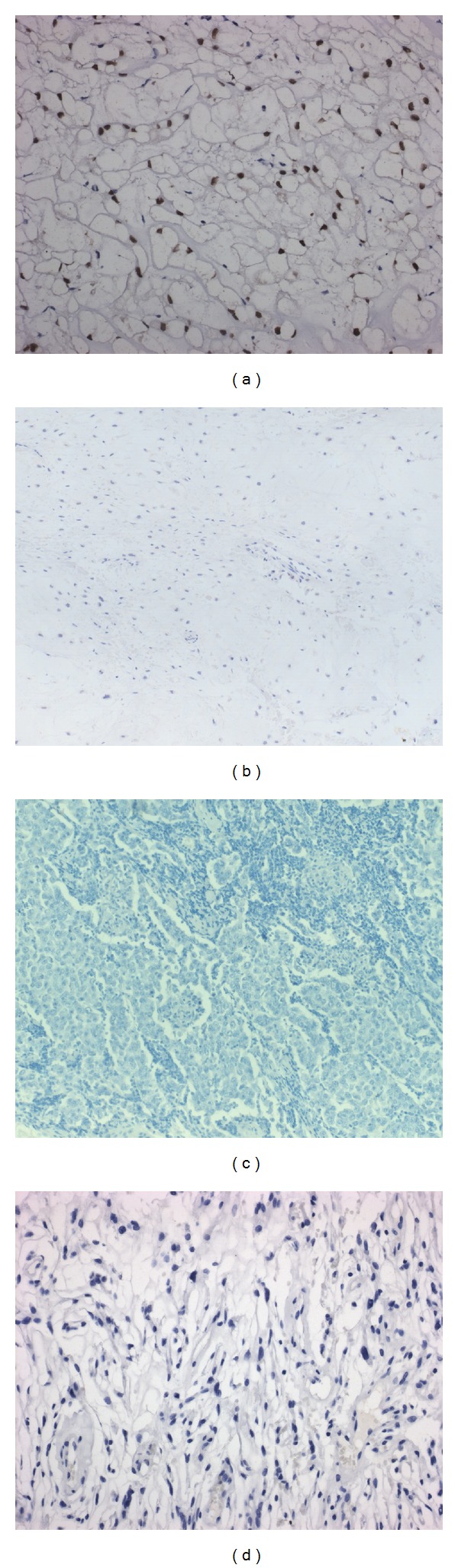
(a) Nuclear staining for brachyury in the neoplastic cells of chordoma (brachyury stain; original magnification, ×200). Absence of stain for brachyury in chondrosarcoma: (b) chordoma (brachyury stain; original magnification, ×100), (c) seminoma (brachyury stain; original magnification, ×200), and (d) chordoid meningioma (brachyury stain; original magnification, ×200).

According to recent evidence, brachyury represents a unique specific diagnostic marker for chordoma, helpful to differentiate this tumour from all of its histological mimickers. Indeed it was shown that most of axial and skull-base chordomas—ranging between 89.7% and 100%, according to the study [4, 8, 9]—including dedifferentiated and metastatic ones display nuclear expression for brachyury (Figure 1(a)) [6–11], with the absence of staining occasionally observed in some conventional and chondroid cases [8, 9] presumably depending upon inadequate fixation in the material and poor antibody penetration [9]. The striking specificity of brachyury stain in the distinction of chordoma from its histological mimickers was demonstrated in a number of studies [6–11]. In detail, no evidence of brachyury expression was reported in chondrosarcoma (Figure 1(b)), liposarcoma, myoepithelial tumors [4], and mucoepidermoid or mucinous carcinoma [8, 12]. Though a focal, weak, immunoreactivity for brachyury was occasionally noted in germ cell tumors, both seminoma and unspecified subtypes [8, 12], no staining for this protein was found in a more extensive study of 111 germ cell tumors, including different subtypes [11], and we also failed to find any brachyury immunohistochemical expression in a small cohort of 10 seminomas (unpublished data) (Figure 1(c)).

The evaluation of brachyury stain may also be helpful in the distinction of chordoma from CCRCC metastatic to the bone. Indeed, no staining for this protein was evidenced in a large series of CCRCC at different sites [11] and in a previous study we found only a focal membranous brachyury staining in one case of CCRCC metastatic to the CNS [26].

The differential diagnosis of chordoma arisen in the intracranial compartment involves chordoid meningioma, a variant featuring chords or trabeculae of eosinophilic, vacuolated cells in a mucoid matrix background [25], and characterized by a high rate of recurrence following subtotal resection [27]. Further, complicating the issue is the similar radiographical imaging of the two entities [6], since meningioma may infiltrate the adjacent bone, and on the other hand, chordoma may invade the dura. In addition, particularly challenging is to differentiate chordoma from primary osseous chordoid meningioma, which does not show any dural connection [28, 29], and chordoid meningioma from primary intradural chordoma, which does not display bone invasion [6, 18, 19]. A panel including EMA, cytokeratins, and S100 protein may be helpful to distinguish the two tumours, as all of the three are positive in chordoma, while chordoid meningioma only features EMA stain. Nonetheless, it may be difficult to settle a definitive diagnosis in small biopsies showing ambiguous staining for these markers since expression of wide spectrum cytokeratins and S100 protein, though focal and weak, was also reported in chordoid meningiomas [7, 10]. Again, the immunohistochemical detection of nuclear brachyury has been demonstrated to be a sensitive and specific marker for chordoma, in the differential diagnosis towards chordoid meningioma [7, 10]. Indeed this protein is expressed in the former—even in pure intradural cases—but not in the latter tumor (Figure 1(d)) [7, 10], including primary osseous chordoid meningioma [28].

Finally, since a positive stain for brachyury was also demonstrated in extra-axial chordomas [12, 30, 31], immunohistochemistry against this protein may be used in order to distinguish chordomas arisen in the soft tissues towards histological mimickers such as myoepithelioma [12, 30, 31], which is of striking importance due to the tendency to grow and recur of the former.

## 3. Brachyury for the Differential Diagnosis of HBL

HBL is defined as a slowly growing, highly vascular tumor, which may occur either sporadically or in the setting of the Von Hippel-Lindau (VHL) syndrome. The latter is an autosomal dominant hereditary neoplasia syndrome which is characterized by germline mutations of the vHL gene and by the predisposition to develop CNS or retinal HBL, renal cell carcinomas and cysts, pancreatic carcinomas and cysts, pheochromocytomas, and epididymal cystadenomas [32]. HBL typically occurs within the CNS, predominantly in the cerebellum and in the spine [33], though supratentorial and meningeal locations have been also reported [34]. Exceptionally, HBL may also occur outside the CNS, in the kidney [35–37], adrenal gland [38], and soft tissues [39], usually as a component of VHL syndrome. This tumour is histologically comprised of stromal cells and small blood vessels [40]. The stromal cells represent the neoplastic component of HBL and are characterized by the presence of numerous lipid-containing vacuoles which give them a clear-cell appearance. Their nuclei may vary in size and occasional atypical and hyperchromatic nuclei and rare mitoses may be observed [40]. Due to the morphological features of the stromal cells of HBL, this neoplasia may mimic other tumors occurring in the CNS, kidney, or soft tissues, as explained in the next paragraphs.

### 3.1. Differential Diagnosis in the CNS

Due to the clear cell morphology of the stromal cells, HBL occurring in the CNS needs to be differentiated from metastatic clear cell renal cell carcinoma (CCRCC). Indeed, HBL and CCRCC may coexist in patients with VHL syndrome, and the synchronous or metachronous presence of HBL and CCRCC metastatic to the CNS has been also reported in these individuals [33]. Even more histologically challenging is the rare occurrence of metastatic CCRCC to a HBL [41, 42].

The distinction between HBL and metastatic CCRC is particularly relevant from therapeutic and prognostic viewpoints. Indeed, HBL is a benign, indolent, WHO grade I tumour [40], treated by surgery alone, while CCRCC metastatic to the CNS carries an adverse prognosis and may need adjuvant aggressive therapies after surgical removal.

Since the distinction of HBL from metastatic CCRCC may be arduous at the histological examination with the only conventional haematoxylin and eosin stain, a number of studies have been carried out in the aim to find immunohistochemical markers able to discriminate between these two entities [26, 43–48]. The diagnostic value of markers such as EMA, cytokeratins, CD10, RCC protein, PAX-2, or PAX-8, which are positive in CCRCC and negative in HBL, may be limited by the possibility of negative metastatic CCRCC cases [43, 47, 49]. On the other hand, the utility of diagnostic markers for HBL, such as D2-40, inhibin-A, and aquaporin-1 [43, 47, 48, 50], may be questioned by the occurrence of positive metastatic CCRCC cases [47, 48, 51].

**Figure 2:**
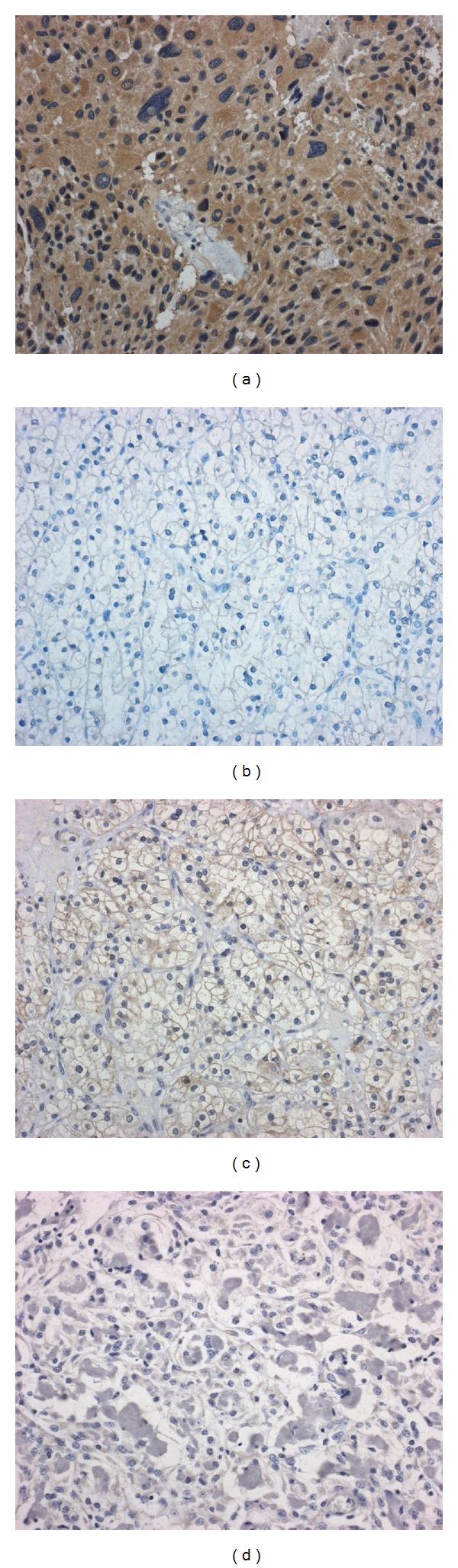
(a) Cytoplasmic staining for brachyury in the neoplastic cells of hemangioblastoma (brachyury stain; original magnification, ×200). (b) Absence of stain for brachyury in CCRCC (brachyury stain; original magnification, ×200). (c) Membranous stain for brachyury in a case of CCRCC (brachyury stain; original magnification, ×200). (d) No evidence of stain for brachyury in clear cell meningioma (brachyury stain; original magnification, ×200).

In a recent study, we showed that the immunohistochemical detection of brachyury is a sensitive method to distinguish between HBL and metastatic CCRCC [26]. Indeed, a cytoplasmic staining for brachyury is evident in the stromal cells of HBL (Figure 2(a)), but not in the clear cells of metastatic CCRCC (Figure 2(b)). Though brachyury antibody may stain the clear cells within some of metastatic CCRCCs, the different pattern of staining, membranous versus cytoplasmic, allows the distinction from HBL (Figure 2(c)).

Apart from metastatic CCRCC, also angiomatous and clear cell histotypes of meningioma may enter the differential diagnosis of HBL involving the CNS, especially in those cases characterized by supratentorial localization [52–54]. In detail, angiomatous is a grade I meningioma variant [25], which is characterized by a predominance of blood vessels over than that of tumor cells [25] and may mimic HBL depending on the prominence of vessels and on the meningothelial aspect of the neoplastic cells [25, 54]. Due to the appearance of the cytoplasm of the neoplastic cells, also clear cell meningioma may simulate HBL at the histological examination [55]. The distinction of angiomatous meningioma from HBL is irrelevant for therapy, as in both of the cases surgery is curative, but the correct identification of HBL is of crucial importance for possible recognition of VHL disease. The differential diagnosis of HBL towards clear cell meningioma may be more significant, as this variant of meningioma is associated with adverse prognosis and increased risk of recurrence [25]. Staining for EMA which is positive in meningioma and negative in HBL is of diagnostic aid, but again, also the immunohistochemical evaluation of brachyury expression is significantly helpful in the differential diagnosis, as no staining for this protein has been demonstrated in these variants of meningioma [26] (Figure 2(d)).

Thus, brachyury may represent a unique marker for the distinction of HBL from its histological mimics. Cytoplasmic staining for brachyury encountered in the stromal cells of this tumor, but not in the clear cell of metastatic CCRC or in the neoplastic cells of angiomatous or clear cell meningioma, may be of relevant help for the differential diagnosis of these entities.

### 3.2. Differential Diagnosis in the Kidney

HBL may rarely occur in the kidney as a sporadic entity. At present, less than ten cases have been reported in the literature [8, 11, 33, 56]. Due to its rarity, primary renal HBL is usually not considered in the differential diagnosis of renal tumours [56]. Thus it may be underrecognized and mistaken for other neoplasias showing a similar morphology, including renal cell carcinoma, adrenal cortical carcinoma, and paraganglioma [33]. Nonetheless, a correct diagnosis is important for patients, as hemangioblastoma is a benign disease, which does not need any further treatment after surgery, unlike malignant CCRCC or adrenocortical carcinoma [56]; even more, a diagnosis of HBL warrants further evaluation for von Hippel Lindau disease. The peculiar clinicopathological features of renal HBL, which affects older individuals and more frequently presents as a solid mass in comparison to its CNS counterpart [37], complicate the differential diagnosis versus other renal tumors. Even more, HBL of the kidney may show a predominance of cells with eosinophilic cytoplasm, and even rhabdoid features, which may suggest a malignant phenotype [37]. The use of an immunohistochemical panel, including EMA, cytokeratins, CD10, inhibin, S100 protein, NSE, and chromogranin has been proposed for the differential diagnosis of renal HBL versus CCRCC, adrenal carcinoma, and paraganglioma. Indeed, EMA, cytokeratins, and CD10 are usually negative in HBL and positive in CCRCC [35], while inhibin, S100 protein, and NSE immunoexpression is found in HBL [37]. On the other hand, a negative staining for chromogranin or synaptophysin generally excludes the hypothesis of adrenal carcinoma and paraganglioma, which stain positively for these proteins [36]. Nevertheless, in some cases the distinction between renal carcinoma and HBL may be challenging even by using immunohistochemistry. Indeed, focal EMA and CD10 stains have been noted in renal HBL [48, 56]; in addition, renal cell carcinoma with rhabdoid features may show diffuse staining for NSE, focal staining for EMA and S100 protein, and reduced cytokeratins expression [57].

**Figure 3:**
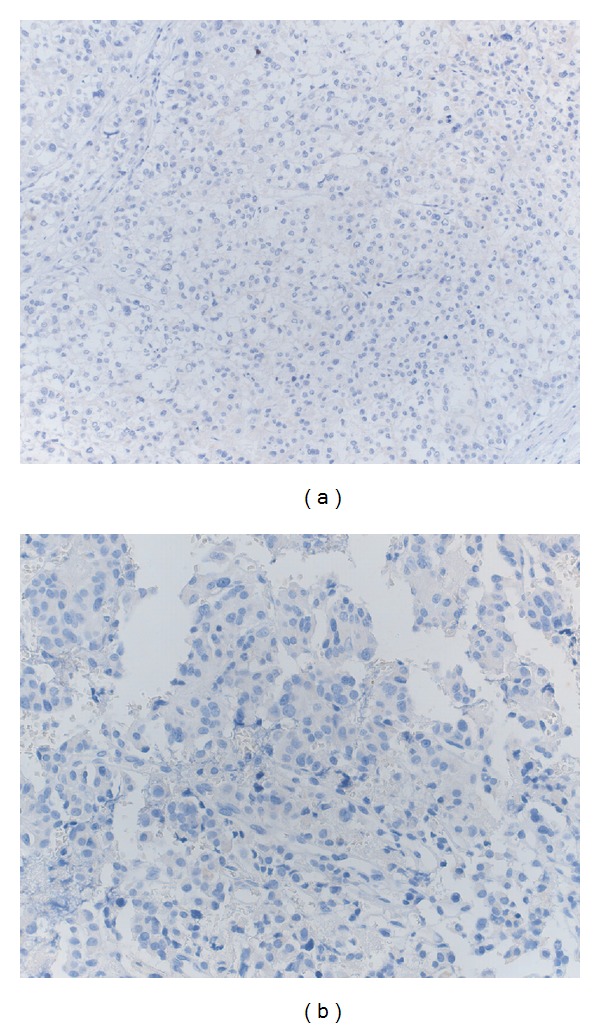
(a) No evidence of brachyury stain in the neoplastic cells of adrenal carcinoma (brachyury stain; original magnification, ×200) and (b) of paraganglioma (brachyury stain; original magnification, ×400).

At present, no data are available on the expression of brachyury in the stromal cells of renal HBL. Nonetheless, renal HBL have been shown to display an immunohistochemical profile equivalent to that of CNS HBL with regard to NSE, inhibin, EMA, cytokeratins, and CD10 [36]. Thus we may speculate that also brachyury expression may be found in these tumors. If so, this marker might be used in the differential diagnosis of histological mimickers occurring in the kidney. Indeed, as previously shown, brachyury stain is negative in primary CCRCC, with a membranous, and not cytoplasmic or nuclear stain, in some cases [26]. In order to analyze whether brachyury may be also used for the differentiation of renal HBL from adrenal carcinoma or paraganglioma, we tested the expression of this marker in five adrenal carcinomas as well as in five paraganglioma (unpublished data) (see [26] for details on immunohistochemical methods). Interestingly, none of the analyzed cases showed any brachyury expression (Figures 3(a) and 3(b)). Therefore, taking into consideration these results, we conclude that brachyury expression may be added to the immunohistochemical panel useful for the differential diagnosis of renal HBL from other neoplastic histological mimickers, such as CCRCC, adrenal carcinoma, and paraganglioma.

## 4. Conclusions

The histological features of chordoma and HBL overlap those of many other tumors arising in the same sites. Recognition of these neoplasias has important, therapeutic, and prognostic, relevance. The use of brachyury stain has been proven to be a unique, highly sensitive, and specific method for the differential diagnosis of chordoma by several studies. Nonetheless, according to our recent findings we suggest that the immunohistochemical evaluation of brachyury is performed in routinary practice also in order to distinguish HBL from histological mimickers occurring in the same sites. Finally, as it was already questioned by Chhieng and Siegal [58], we wonder whether it is still correct to define brachyury as a marker of notochordal differentiation, seen its expression also in the stromal cells of HB.
